# Numbers of wildlife fatalities at renewable energy facilities in a targeted development region

**DOI:** 10.1371/journal.pone.0295552

**Published:** 2023-12-15

**Authors:** Tara J. Conkling, Amy L. Fesnock, Todd E. Katzner

**Affiliations:** 1 Forest and Rangeland Ecosystem Science Center, U.S. Geological Survey, Boise, Idaho, United States of America; 2 Desert District Office, U.S. Bureau of Land Management, Palm Springs, California, United States of America; MARE – Marine and Environmental Sciences Centre, PORTUGAL

## Abstract

Increased interest in renewable energy has fostered development of wind and solar energy facilities globally. However, energy development sometimes has negative environmental impacts, such as wildlife fatalities. Efforts by regional land managers to balance energy potential while minimizing fatality risk currently rely on datasets that are aggregated at continental, but not regional scales, that focus on single species, or that implement meta-analyses that inappropriately use inferential statistics. We compiled and summarized fatality data from 87 reports for solar and wind facilities in the Mojave and Sonoran Deserts region of southern California within the Desert Renewable Energy Conservation Plan area. Our goal was to evaluate potential temporal and guild-specific patterns in fatalities, especially for priority species of conservation concern. We also aimed to provide a perspective on approaches interpreting these types of data, given inherent limitations in how they were collected. Mourning doves (*Zenaida macroura*), Chukar (*Alectoris chukar*) and California Quail (*Callipepla californica*), and passerines (*Passeriformes*), accounted for the most commonly reported fatalities. However, our aggregated count data were derived from raw, uncorrected totals, and thus reflect an absolute minimum number of fatalities for the monitored period. Additionally, patterns in the raw data suggested that many species commonly documented as fatalities (e.g., waterbirds and other nocturnal migrants, bats) are rarely counted during typical pre-construction use surveys. This may explain the more commonly observed mismatch between pre-construction risk assessment and actual fatalities. Our work may serve to guide design of future scientific research to address temporal and spatial patterns in fatalities and to apply rigorous guild-specific survey methodologies to estimate populations at risk from renewable energy development.

## Introduction

Increased interest in renewable energy as a tool to address climate change and meet growing demand of the global energy market has fostered rapid development of wind and solar energy facilities both in the United States and around the world. As a result, renewable energy development is rapidly expanding to meet increased demand without increasing CO_2_ emissions. Since 2009 in the U.S.A., growth rates of installed capacity of utility-scale wind and solar energy exceed 300% and 9400% respectively [1–3].

Renewable energy development often results in some level of negative environmental impact, notably habitat loss and fragmentation, along with fatalities of birds and bats [4–6]. These fatalities are largely believed to be caused by collisions with turbines, photovoltaic panels, and heliostat solar reflectors, or other facility infrastructure (e.g., perimeter fences, gen-tie and associated transmission lines). However, birds may also be killed at solar facilities by unintentional grounding or singeing from the concentrated beams of sunlight at CSP power towers, and both birds and bats have been documented drowned in wastewater evaporation ponds found at concentrated solar power (CSP) facilities or been inadvertently trapped in facility buildings and equipment [7–12]. Consequently, there is substantial interest in finding tools to balance the competing interests of maximizing energy production potential and minimizing fatality risk to both local and migratory wildlife species. To do this though, first, developers and land management agencies need to identify the potential avian and bat species at risk and the numbers of individuals of each species found dead at these facilities.

Current research to describe impacts of renewable energy on wildlife often focuses either on single species or taxa (e.g., [13–15]) or on meta-analyses that summarize and analyze fatality estimates generated across a suite of individual environmental reports or datasets [7, 10, 16, 17]. Alternatively, they may rely on pre-construction risk assessment use surveys to estimate fatality risk at a given location [18, 19]. However, there are limitations to inference from these approaches [12]. For example, these assessments are biased towards facilities with publicly available data or where authors have sole access to confidential reports. In fact, none of these published meta-analyses that summarized data from environmental reports from wind or solar facilities have either systematically or randomly sampled the facilities that were included in analyses. As a consequence, even the most complete compendia [e.g., 7, 16] omit many reports that are not publicly available and, thus, the level of inference of their analyses is constrained. Similarly, the field survey techniques used to generate fatality data often are inconsistent among reports or facilities. As such, use of inferential statistics to estimate pooled fatality rates is problematic [20, 21]. Additionally, pre-construction surveys are often designed to meet state or federal monitoring guidelines or requirements (e.g. Environmental Impact Reviews (EIR) or Statements (EIS)), rather than defined research objectives, and surveys may be limited to focal species groups (e.g. raptors) or be spatially or temporally limited (e.g., breeding season point counts), all factors that can reduce the applicability of pre-construction monitoring data to assess post-construction fatality risk [18, 22]. Finally, meta-analyses often group species into broad categories (e.g., raptors, waterbirds, passerines), may ignore some taxa altogether (e.g., bats) and do not identify individual facilities. This approach is a useful data visualization tool for pooled data, but it can obscure important temporal, spatial, and taxon- or species-specific patterns of substantial interest, especially for threatened or endangered species.

Within the U.S.A., regional land managers and regulators are tasked to use the “best available science” to make permitting and mitigation decisions for renewable energy facilities [23]. However, the substantial limitations of existing studies and reports as outlined above can obscure that science. In particular, existing data on wildlife fatalities at renewable energy facilities within regions are rarely consolidated into a single data repository and may be available only as difficult-to-interpret single-species studies, large meta-analyses, consultant reports, or a widely dispersed set of datasheets reported to multiple agencies by wildlife consultants.

The objective of this study was to address this problem by compiling and summarizing data on fatalities from renewable energy facilities in one region of the U.S.A. that is a focus for renewable energy development. We evaluated fatality data from parts of the Mojave and Sonoran Deserts of southern California that are within the Desert Renewable Energy Conservation Plan (DRECP) area [24]. Our goals were (1) to quantify numbers of fatalities of birds and bats counted at renewable energy facilities in the vicinity of the DRECP, (2) to examine the dataset to identify potential temporal and taxonomic-specific patterns in fatalities, especially for priority species of conservation concern, and (3) given the variability and inconsistency among strategies and availability of reports, to provide a perspective on interpreting these fatality data and the caveats that might accompany those interpretations. By presenting trends in the raw data, we hope to broadly describe an appropriate frame of reference for inference about numbers of species-specific fatalities at these facilities and to provide a starting point for subsequent studies with robust experimental design that can lead to stronger inference.

## Materials and methods

### Study area

The DRECP was approved in 2016 by the California Energy Commission (CEC), California Department of Fish and Wildlife (CDFW), Bureau of Land Management (BLM), and U.S. Fish and Wildlife Service (FWS) to identify areas in the region that may be appropriate for utility-scale renewable energy development, to facilitate the application process for renewables, and to manage long-term conservation in the region. Land cover in the Mojave desert is scrub dominated by creosote bush (*Larrea tridentata*), Joshua tree (*Yucca brevifolia*), and number of cactus and succulent plants adapted to desert habitat. Land cover in the Sonoran Desert is typified by cacti, especially saguaro (*Carnegiea gigantea*) and cholla (*Cylindropuntia spp*.), but also includes species found in the Mojave desert. Topography in both habitats is rugged with steep mountains and hillsides punctuated by alluvial flats. The climate in both regions is exceptionally arid, although the Sonoran desert is unique in having two seasonal monsoons [25]. The DRECP is a focus for renewable energy development and there is substantial management interest in understanding potential impacts to wildlife from this development. As such, post-construction monitoring is more regular in the DRECP than in many other regions of the USA, making it a good site for the evaluation we performed here.

### Data collection and analyses

We use three methods to obtain data on fatalities at renewable energy facilities within the DRECP boundary for the time period ranging from the first installation of wind turbines in the Tehachapi Pass in the early 1980s through December 2019. First, we used online search engines to search the internet for environmental reports that had been posted online. Second, we searched publicly available document collections and California-specific public databases (e.g., American Wind Wildlife Institute documents library (https://awwic.nacse.org/), California Energy Commission) to identify environmental reports they contained. Finally, we queried databases at federal, state, and county-level agencies for environmental reports not collected in our other searches. We focused especially on gathering unpublished environmental reports, usually by consultants (hereafter, “consultant reports”), containing wildlife survey data from proposed and operational wind and solar energy facilities located within or closely adjacent (<20km) to the DRECP boundary. Many of the reports published prior to 2018 we accessed have also been summarized in a previous review of the effects of renewable energy on birds and bats [12]. We also searched the sources listed above for data available from peer-reviewed published literature that included species-specific fatality totals that were not reported elsewhere.

As part of our third search method outlined above, we included consultant reports provided to FWS and BLM, as well as raw data for fatalities and injured birds found incidentally or during systematic surveys provided in the form of spreadsheets required under Special Purpose Utility Permits (SPUT) issued to energy facilities (hereafter, “SPUT reports”). These permits authorized the electrical utilities to collect and temporarily possess migratory bird carcasses found at facilities. SPUT reports are only required to document avian fatalities, not bats. However, if the authors voluntarily included bat fatalities in their report, we included those numbers in our subsequent data summary. All data were from facilities located on public lands, primarily BLM administered lands.

For each consultant or SPUT report, we documented the facility name, energy source and technology type (e.g., wind, solar photovoltaic (PV), concentrating solar power (CSP) parabolic troughs, CSP power tower) and the construction periods (pre- or post- construction). We also summarized the start and end dates of the surveys, the type of survey data collected (e.g., fatality surveys, incidental reporting), and details of the survey methodologies. Finally, we recorded detections of wildlife fatalities, their date, and, where noted, the type of infrastructure with which the fatality was associated (e.g., sometimes fatalities are associated with buildings, fences, or power lines at facilities, rather than the electrical generating infrastructure itself). Although we collected data on both fatalities and injured birds and bats, our subsequent analyses only included individuals found dead, or who later died as a result of their initial injuries.

In some cases, data were summarized across seasons or annual reporting periods and were thus not suitable for subsequent within-season or period analysis. Some facilities had multiple reports for overlapping periods of time (e.g., we obtained both annual and monthly summary reports for the same year). To avoid double-sampling, we excluded those reports that spanned the shorter monitoring periods. Additionally, sometimes the monitoring dates and associated raw fatality data available in SPUT reports overlapped the time period for a given consultant report. In those cases, we preferred to use the individual observations available in the SPUT data, as they tended to have more precise temporal information than did the consultant reports (i.e., they usually specify the date and location for each individual carcass, whereas the consultant reports typically aggregate data across periods or taxa).

We compiled the numbers of raw “uncorrected” fatalities documented in consultant and SPUT reports into summary tables by energy type (e.g., solar or wind), with fatality totals grouped by species and summed by individual years, across all years monitored, and at each energy facility. These raw fatality totals were not adjusted for factors such as searcher efficiency or carcass persistence that can negatively influence detection probabilities [20] We also calculated the proportion of total fatalities comprised by each species or species group. We summarized these annual fatality totals in an uncorrected manner (e.g., totals were not weighted by search frequency or seasonal differences in survey effort) across all monitoring periods with available data. Our subsequent analyses focused on the uncorrected fatality survey totals for a number of federal and state-listed species of conservation priority (hereafter “focal species”). In the DRECP, these were willow flycatcher (*Empidonax traillii*), least Bell’s vireo (*Vireo bellii pusillus*), bank swallow (*Riparia riparia*), western yellow-billed cuckoo (*Coccyzus americanus occidentalis*), Gila woodpecker (*Melanerpes uropygialis*), northern flicker (*Colaptes auratus*), burrowing owl (*Athene cunicularia*), and Swainson’s hawk (*Buteo swainsoni*). We also summarized cumulative fatality totals for several focal species groups that can be difficult to differentiate in remains, including rails (*Rallidae*), thrashers (*Mimidae*), and warblers (*Parulidae*). Finally, in addition to fatality summary tables, we summarized data in the SPUT reports by month to evaluate temporal patterns of fatalities. We did not include the consultant reports in these monthly summaries because those reports only sometimes contained temporal data at the scale needed for this analysis.

While these reports typically are designed to meet guidelines or requirements of state and federal environmental reviews (e.g., environmental impact statements or reviews; EIS or EIR), they seldom implement experimental study designs, thereby restricting the inferences across facilities [12]. For example, most consultant reports with fatality monitoring data calculated an index of fatalities relative to nameplate capacity of a given facility (i.e., fatalities/MW) to standardize rates relative to other locations. However, this metric does not necessarily account for variation in mortality rates resulting from factors such as season, geographic region, turbine characteristics (e.g., rotor-swept area, hub height, blade length), turbine operational status (e.g., curtailment periods, non-operational (broken) turbines, rotations per minute) [26], technology type (e.g., Solar PV, CSP parabolic trough, CSP Power Tower, Wind), or variation in survey efforts (e.g., size of search area, frequency of searches, use of detection dogs versus human searchers whether or not surveys accounted for detection probability). Additionally, adjusted fatality estimates that accounted for survey biases in searcher efficiency or carcass persistence in the landscape were commonly calculated only for broad taxonomic groupings (e.g., passerines, water-associated birds, bats) or size categories (e.g., small birds, medium birds, large birds), rather than for individual species, limiting our ability to compare species-specific fatality estimates among facilities. Furthermore, some facilities in our dataset only reported incidental observations. As such, to gain the broadest inference from all documented fatalities, we ignored corrections for survey bias, and we report raw totals to represent the minimum number of fatalities at a given location. This is because it would be misleading and statistically inappropriate to apply inferential statistics to the cumulative dataset of fatality estimates or likewise to directly interpret data patterns across facilities for data of varying rigor. However, although these minimum totals may not be fully equivalent across facilities due to methodological differences such as sampling duration or survey effort, these pooled data can still provide general information about species detected as fatalities, temporal patterns, and fatalities among types of renewable energy.

## Results

**Fig 1:**
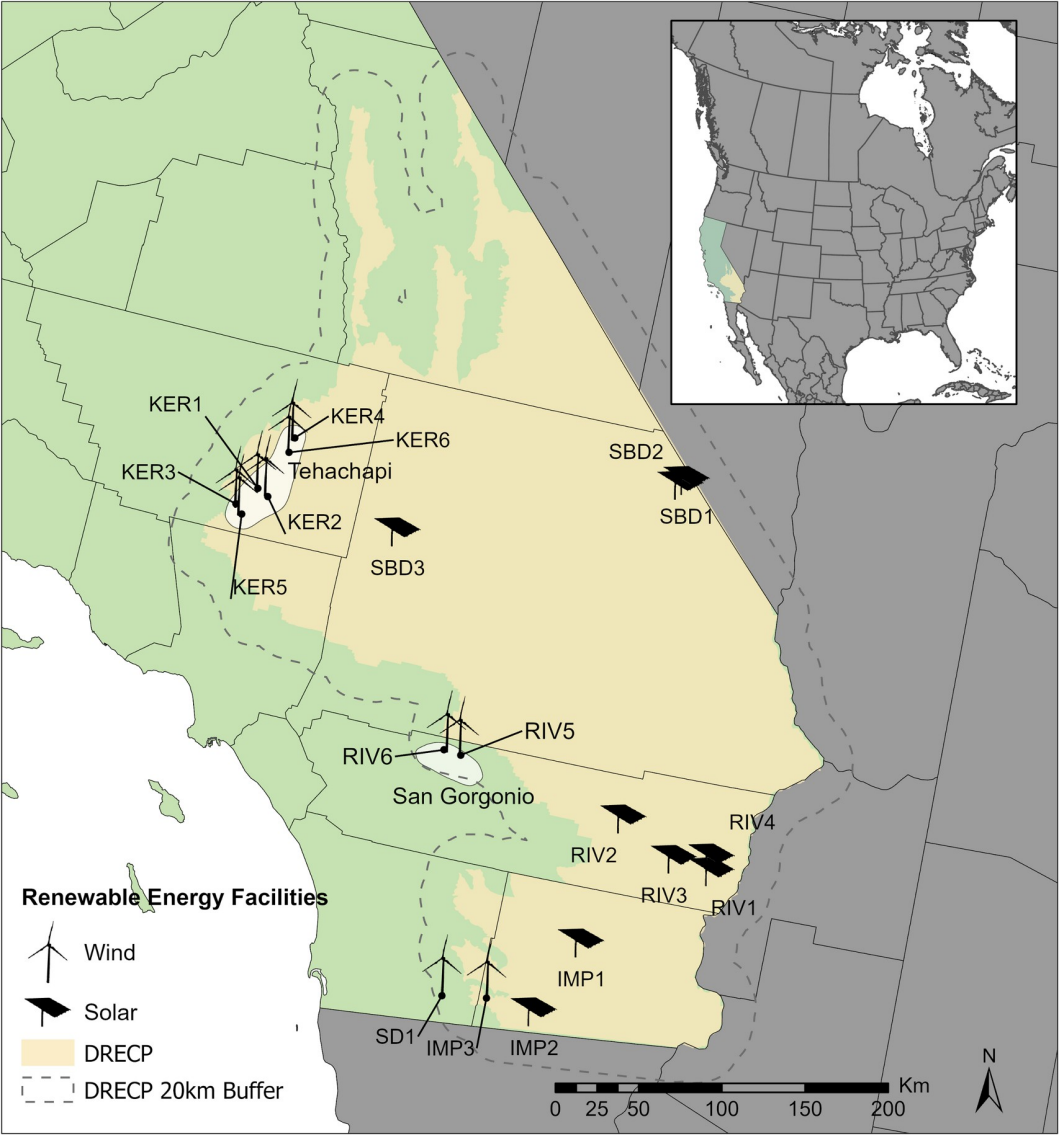
Location of wind and solar facilities used to assess results from fatality surveys at facilities within and in close proximity to the Desert Renewable Energy Conservation Plan Area (“DRECP” in blue) in California, U.S.A. (in yellow). Facilities are given names that show the county in which they are located (e.g., KER1 is in Kern County, SBD1 is in San Bernardino County, RIV1 is in Riverside County, IMP1 is in Imperial County, and SD1 is in San Diego County). Also shown in white are the locations of the Tehachapi and San Gorgonio Wind Resource areas. Basemap made with Natural Earth (www.naturalearthdata.com). DRECP boundary obtained from https://drecp.databasin.org/datasets/b1eb4709a1eb4f6db1dfe7dd5479f6c9/[24].

We obtained 87 consultant reports and, after excluding duplicated datasets, we evaluated information from 64 reports on fatality surveys at 18 facilities (11 wind, 7 solar) conducted between 1996 and 2019. In these reports there were documented 262 species or species groups and 4757 fatalities that were not listed in SPUT reports. We also considered data in SPUT reports from 10 facilities (3 wind, 7 solar), including 3 (2 solar, 1 wind) for which there were no available consultant reports (Tables 1 and S1 and S2 and Fig 1 and S1 Text). In the SPUT reports (S1 and S2 Tables) there were 3326 documented fatalities from 223 species or species groups. Some, but not all, of these data in SPUT reports were originally mentioned in the consultant reports. Data in these reports are provided in the Supporting Information (S1 and S2 Tables).

**Table 1:** Availability of fatality data from unpublished reports or SPUT datasheets by energy facility.

			Report	SPUT
Facility	Type	County	Monitoring Dates	Monitoring Dates
**Solar**				
IMP_1	PV	Imperial	6–10/2016	‐‐
IMP_2	PV	Imperial	10/2014–9/2016	‐‐
RIV_1	PV	Riverside	3-5/2016	1/2016–12/2019
RIV_2	PV	Riverside	8/2011–10/2014	9/2011–12/2019
RIV_3	CSP Trough	Riverside	3/2015–2/2017	3/2015–12/2019
RIV_4	PV	Riverside	‐‐	3/2016–4/2019
SBD_1	PV	San Bernardino	‐‐	4/2014–12/2019
SBD_2	CSP Tower	San Bernardino	11/2013–10/2015	11/2011–7/ 2018
SBD_3	CSP Trough	San Bernardino	10/2013–10/2015, 3/2016	10/2013–12/2019
**Wind**				
IMP_3	HAT	Imperial	3/2018–3/2019	1/2013–12/2015
KER_1	HAT	Kern	6/2009–5/2010	‐‐
KER_2	HAT	Kern	3/2011–5/2019	‐‐
KER_3	HAT	Kern	3/2013–2/2015	‐‐
KER_4	HAT	Kern	1/2013–1/2017	‐‐
KER_5	HAT	Kern	‐‐	1/2015–12/2015
KER_6	HAT	Kern	6/2009–6/2010, 8/2011–6/2013	1/2017–12/2019
Tehachapi	HAT	Kern	10/1996–5/1998	‐‐
RIV_5	HAT	Riverside	3/2008–3/2009	‐‐
RIV_6	HAT	Riverside	8/1995–8/2000, 8/2009–8/2014	‐‐
San Gorgonio	HAT	Riverside	3/1997–5/1998	‐‐
SD_1	HAT	San Diego	1/2006–1/2007	‐‐

Facilities are codes by county, types are solar photovoltaic (PV), concentrating solar power trough or tower (CSP Trough or Tower), and wind turbines, which were solely horizontal axis turbine (HAT). Data could be available either as a consultant report (Report) or a spreadsheet in a Special Purpose Utility Taking Permit (SPUT). “Monitoring Dates” indicates whether or not that data type was available, and dates are given as month/year.

Data were collected in all months of the year, but only 76% of the facilities conducted mortality surveys for 12 continuous months (Table 1). Additionally, 33% (*n* = 7) of these facilities conducted surveys in the same month more than once, while 4 other facilities compiled reports for multiple years, but solely documented fatalities discovered incidentally, rather than during systematic surveys. For facilities from which SPUT reports were available, nearly all (90% or 9 facilities and 3326 observations) reported fatalities across multiple years, although these observations included both survey and incidentally found carcasses.

### Trends in fatalities of birds and bats at DRECP solar facilities

**Fig 2:**
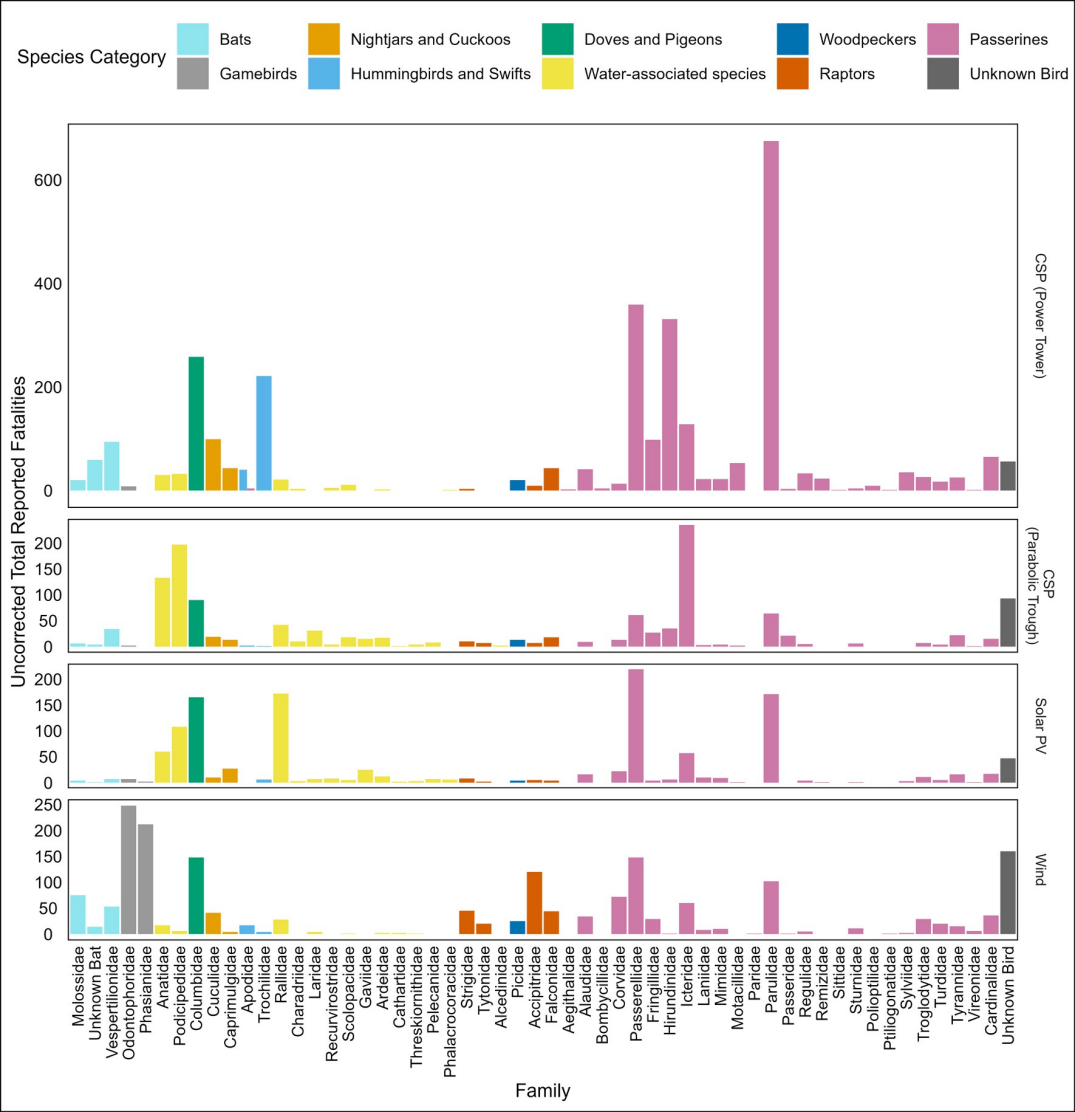
Total raw, uncorrected counts of bird or bat carcasses (n = 8054; grouped by taxonomic family), documented in consultant and SPUT reports from renewable energy facilities in the Desert Renewable Energy Conservation Plan Area. Plots are by facility subtype (e.g., Solar PV, Concentrating Solar Power (CSP) parabolic trough, CSP Power Tower, and Wind). Plot color is indicative of the broad species groups commonly used in meta-analyses (e.g., [7, 16]) that merge multiple Orders and Families.

The species most commonly reported as found dead at solar facilities was mourning dove (*Zenaida macroura*; *n* = 355 carcasses; S1 Table). Yellow-rumped (*n* = 256; *Setophaga coronate*) and yellow warblers (*n* = 180; *Setophaga petechia)* were the next most commonly found species. Brown-headed cowbird (*n* = 155; *Molothrus ater*), eared grebe (*n* = 153; *Podiceps nigricollis*), white-crowned sparrow (n = 137; *Zonotrichia leucophrys*), Wilson’s warbler (*n* = 133; *Cardellina pusilla*), greater roadrunners (*n* = 126; *Geococcyx californianus*) and American coot (*n* = 124; *Fulica americana*) were the next most common species identified. Considering both SPUT and consultant reports together, passerines accounted for nearly 30% of all reported species and 60% of all uncorrected observations (*n* = 3522 passerines) (S1 Table). However, these data were dominated by a few families (Fig 2), primarily warblers (*n* = 905; *Parulidae*) and sparrows (*n =* 638; *Passerellidae*), blackbirds (*n* = 386; *Icteridae*), and swallows (*n* = 371; *Hirundinidae*). Large numbers of carcasses were reported as being of unknown species (n = 12 76; 22%), including 194 classified only as “unknown bird”.

Individual focal species and one focal group (thrashers) were rarely reported found at solar facilities (again considering both types of reports; S1 Table). The most common of these were northern flicker (*n* = 22 carcasses), burrowing owl (*n* = 11), bank swallow (*n* = 9), and Crissal thrasher (*n* = 3; *Toxostoma crissale*). However, there were large numbers of grebes and rails, as noted above, as well as 77 sora (*Porzana carolina*) and 14 common gallinules (*Gallinula galeata*). Likewise, as noted above, warblers were reported in very large numbers; these totals include a large number of unknown warbler species (*n* = 94), and >15 fatalities of six other warbler species. Interestingly, the warblers and hummingbirds were more commonly found at the single CSP facility, and the waterbirds were more commonly found at the PV and solar trough facilities (Fig 2).

Bats were also rarely reported found at solar facilities (n = 149), with Canyon Bats (*Parastrellus hesperus*) accounting for 26% (n = 39) of these carcasses. However, in some cases, reports only documented avian fatalities or did not identify bat fatalities to species. In addition, SPUT reports are only required to document avian fatalities, so these totals reflect a minimum number of bats found at solar facilities.

### Trends in fatalities of birds and bats at DRECP wind facilities

**Fig 3:**
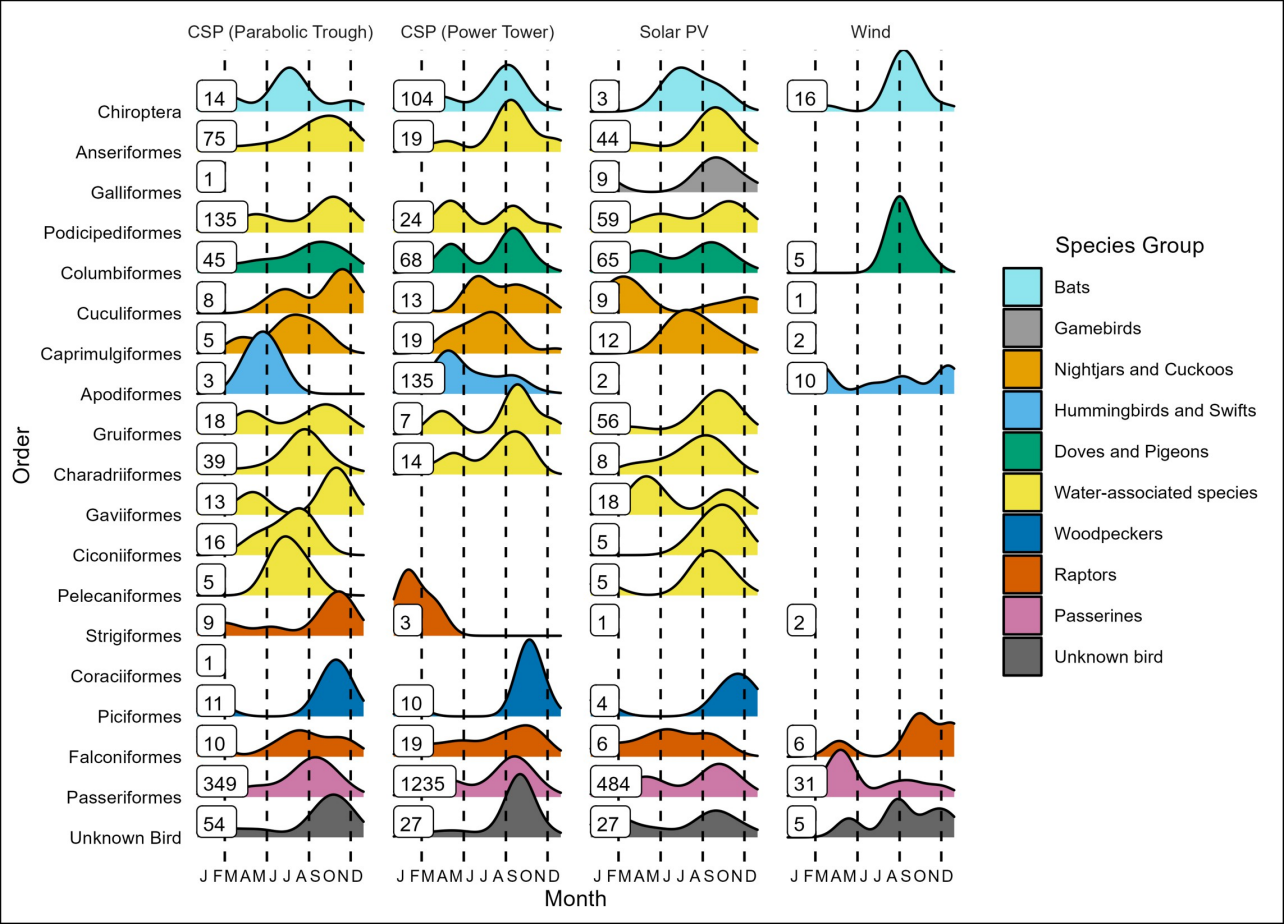
Ridgeplot of the temporal distribution of raw, uncorrected counts of bird or bat carcasses (n = 3304; grouped by taxonomic order), documented in SPUT reports from renewable energy facilities in the Desert Renewable Energy Conservation Plan Area (DRECP). Plots are by month and facility subtype (e.g., Solar PV, Concentrating Solar Power (CSP) parabolic trough, CSP Power Tower, and Wind). Plot color is indicative of the broad species groups commonly used in meta-analyses (e.g., [7, 16]) that merge multiple Orders. Number of reported fatalities is shown with each plot (no plots were generated for ≤ 2 fatalities).

California quail (*n* = 236; *Callipepla californica*) and chukar (*n* = 212; *Alectoris chukar*) were the most common species reported dead in both types of reports from wind facilities (S2 Table). At an order level, Passeriformes (*n* = 725) and Galliformes (*n =* 460) comprised 58% of documented bird fatalities (*n =* 2043; Fig 3). Beyond the many carcasses that could not be identified, other common species found dead included mourning dove (*n* = 89) and red-tailed hawk (*n* = 79; *Buteo jamaicensis*), western meadowlark (*n* = 50; *Sturnella neglecta*), rock pigeon (*n* = 50; *Columba livia*), dark-eyed junco (*n* = 45; *Junco hyemalis*), and greater roadrunner (*n* = 41).

Only three focal species and one species from a focal group (i.e., thrashers) were found dead more than twice at wind facilities. These included northern flicker (*n* = 21), burrowing owl (*n* = 5), ash-throated flycatcher (n = 3; *Myiarchus cinerascens*), California thrasher (*n* = 7; *Toxostoma redivivum*). There were no focal rails found dead at wind facilities (Figs 2 and 3). American Coot (*n* = 25; *Fulica americana*) and Sora (*n* = 3; *Porzana carolina*) and other water-associated species (*n* = 34; e.g., Podicipediformes, Anseriformes, Gruiformes, Gaviiformes) accounted for only 3% of total reported fatalities. Among passerine families, sparrows (*n = 146*) and warblers (*n* = 102) were more commonly found at wind facilities than other groups. The most frequently found warblers included yellow-rumped (*n* = 19), Wilson’s (*n* = 18), and orange-crowned (*n* = 13; *Leiothlypis celata*) warblers. Additionally, bat fatalities accounted for nearly 7% (*n =* 142) of the overall documented fatalities at wind facilities, with the most common species including Mexican free-tailed bats (*n =* 47; *Tadarida brasilensis*) and Hoary bats (*n =* 37; *Lasiurus cinereus*).

### Temporal patterns in fatalities at renewable facilities within the DRECP

Considering only data in SPUT reports, bird and bat fatalities were reported across all months at both wind and solar facilities. However, most incidents occurred during migration periods, especially at solar facilities, with nearly half of fatalities (48%; *n* = 1580) reported during fall (September-November). That said, temporal patterns varied by Order and broad species groups (Fig 3). For example, migrant waterbirds (e.g., Podicipediformes, Anseriformes, Gruiformes, Gaviiformes) and passerines (Passeriformes) were predominantly found during migration periods, while fatalities of raptors (e.g., Falconiformes, Strigiformes) occurred across all seasons. In contrast, fatalities of Caprimulgiformes primarily occurred during the summer breeding season. For the limited SPUT-reported bat fatalities (*n* = *33)*, most incidents at wind facilities occurred during fall migration involving migratory species (e.g., Mexican Free-tailed bats), whereas incidents at solar facilities were primarily resident bat species (e.g., Canyon bats).

## Discussion

The data in these reports are, to our knowledge, the best publicly available dataset to describe species-specific patterns in fatalities at renewable energy facilities within the DRECP. That said, there are several issues regarding study design and data quality that affect inference based on these data (see [12] for documentation of these patterns). As such, these data must be interpreted with caution, with few statistical analyses and from a qualitative perspective. Here we provide a perspective on interpreting them and the caveats that might accompany those interpretations.

The most important caveat is that the aggregated count data we report were not the result of random or systematic sampling of renewable facilities and they were not conducted in a manner that was standardized across all facilities. For example, differences in survey methodologies, (e.g. frequency of survey visits, time of year, size of the search area, use of dogs to search for carcasses) can all influence the number of carcasses found. Likewise, because searcher efficiency and scavenger removal rates for dead birds and bats (to correct raw totals for individuals killed but not detected by searchers on subsequent surveys) were only sometimes estimated, those fatality data are not useful for comparison across all sites [12]. As such, it would be inappropriate to use inferential statistics to analyze aggregated data or to interpret patterns in fatality estimates across facilities [27]. Thus, even straightforward comparison of count data must be done with extreme caution and these totals should be interpreted to reflect *the absolute minimum number* of fatalities at a given location during the monitored time period. Given these caveats, these data can still provide some insight into patterns of species detected as fatalities and about differences among facilities and among technology types. Identification of these patterns also may serve as a starting point for subsequent studies with robust experimental design that can lead to stronger inference.

Waterbirds (e.g., teal (*Anas spp*.), mallard (*Anas platyrhynchos*), grebes (*Podicipedidae*), loons (*Gavia spp*.), phalaropes (*Phalaropus spp*.), ruddy duck (*Oxyura jamaicensis*), spotted sandpiper (*Actitis macularius*)), and forest-nesting warblers (orange-crowned, yellow-rumped, Townsend’s (*Setophaga townsendi*), Wilson’s) both were common in the list of fatalities (S1 and S2 Tables). However, these taxonomic groups were rarely counted on pre-construction use surveys or point counts for live birds documented in reports we surveyed at solar facilities (S1 Text, TJC unpublished observations). This is notable and suggests the weak relationship between risk assessment pre-construction and actual fatalities post-construction at wind facilities also applies to solar facilities.

Although formal statistical comparison to detect trends would be inappropriate, existing data may provide sufficient insight to guide design of follow-up studies that could explore these patterns and to develop rigorous taxon-specific survey methodologies to estimate populations at risk. While it is possible that there were real biological difference in species present during the two construction phases, at face value, that seems unlikely. In fact, the type and quality of data collected often differs between pre- and post- construction surveys, with pre-construction surveys only rarely incorporating any bias-corrections to account for detection probabilities [12]. Similarly, data often are collected at different spatial and temporal scales, such that pre-construction use surveys monitor a large potential facility site, but post construction surveys focus only on a smaller project footprint or individual facility components (e.g., turbines).

Survey methods at pre-construction sites also may not have been appropriate for the species at risk of fatality (e.g., monthly point counts for cryptic or rare species), or they may have been conducted at the wrong time of year or time of day to detect a given species (i.e., surveying during the nesting season for a species only present during migration, or surveying during the day for a nocturnally migratory species). As such, these surveys can only observe species that are physically present during the defined pre-construction survey period, while carcass searches provide evidence that a species was present at a given location (regardless of whether the observer was present when the fatality occurred). For example, although both waterbirds and warblers are nocturnal migrants [e.g., 28, 29] of the two, only warblers typically stopover in desert habitats that lack bodies of water that are necessary for many waterbirds (e.g., grebes, loons) to initiate flight behaviors. As a result, migration surveys or point counts conducted during daylight hours may be effective to detect warblers but not waterbirds. However, as point counts for live birds are designed to detect vocalizing songbirds during the breeding season, this survey technique may not be as effective during migration and winter months. Use surveys that are effective throughout the year, that detect nocturnally migrating passerines, and that can include replication over multiple years, may be appropriate as follow-ons to the existing work. Furthermore, radar, radio-telemetry arrays, or other similar tools [e.g., 30–32] may provide additional insight into flight patterns and behaviors of migratory species that cross over a proposed solar facility, that may be at risk, or that may not be detected during daytime surveys. All of these factors can cause discrepancies between species and numbers found pre- vs post-construction.

Comparison of fatalities among sites and among renewable energy technologies is difficult with these data of varying quality. That said, there are some patterns that may merit future study that can be gleaned from the cumulative dataset. For example, few waterbirds but many raptors were reported dead at wind facilities, but the opposite pattern was noted at solar facilities (many waterbirds, few raptors) (Fig 2). Also, it is noteworthy that at solar facilities, waterbird fatalities were reported at some PV and CSP solar trough facilities, with causes of death due to collisions with panels, unintentional grounding, or drowning in the wastewater evaporation ponds, but fatalities only rarely reported at the CSP tower facility (i.e., Solar_SBD_2; Tables 1 and S1). The opposite pattern was true for warblers and hummingbirds, with most fatalities detected at the single CSP tower (Fig 2), likely due to feather singeing from the concentrated sunlight beams. Additionally, for most of these species, fatalities occurred primarily during migration periods, highlighting the risk these facilities may pose when located in the vicinity of major migration flyways, including the Pacific Flyway that includes southern California (Fig 3; also see [33]). It also is noteworthy that greater roadrunners were found in large numbers at both solar and wind facilities, but it is unclear what ecological factors may be increasing fatalities for this species [34]. Finally, it would be valuable to focus future work to see if these patterns hold up more broadly across greater numbers of facilities. If these patterns were also to be observed in robustly designed studies, then it would be possible to evaluate temporal and spatial patterns in fatalities relative to known migration timing, corridors, and landscape features.

A weakness of existing reporting is that often there is insufficient information in publicly available consultant reports to associate species-specific fatalities with the corresponding infrastructure or seasonal timing of deaths (also see [12]). For example, reports typically document fatalities, not only at wind turbines or solar panels, but also in the vicinity of transmission lines, perimeter fences, and evaporation ponds. However, this detail is commonly summarized only for broad taxonomic groups or across the entire monitoring period, an approach which can obscure temporal, spatial, or species-specific patterns in fatalities. Data on the location of fatalities at solar facilities (e.g., at the power block, fence, gen-tie line, road, pond) are often provided with carcass recovery dates in SPUT forms used by consultants. Incorporating this information into species-specific analyses may be a useful tool to examine within-site trends in fatalities. If additional information was provided on when and where carcasses were found, it would allow managers to better understand species-specific differences in causes of death at these facilities. This in turn could guide future efforts to standardize monitoring and improve fatality mitigation practices at facilities and associated infrastructure buildout, such as transmission lines [35]. For example, the large number of Galliform birds that die at wind facilities (Fig 2) is unexpected since these birds tend to fly at relatively low altitudes above ground. Research from other areas suggests ptarmigan, *Lagopus spp*. and other grouse species die from collision with large monopoles, rather than from impact by turbine blades [36]. More detailed information on locations of fatalities of the Galliformes we note here would provide insight into if they may have died in a similar manner.

Finally, our study emphasizes the importance of applying best management practices for study design, utilization, and data aggregation and dissemination of pre- and post-construction monitoring data [12], especially within regions such as the DRECP that have been prioritized for renewable energy buildout. Given that there is a known emphasis on future development in the DRECP, establishing region wide research objectives, standardized survey methodologies, and improving overall data sharing and aggregation would improve our understanding of fatality patterns and provide guidance retarding effective mitigation practices for affected species.

## Conclusions

There are many anthropogenic sources of bird and bat mortality [11, 37–39]. As renewable energy becomes increasingly more abundant, there is growing interest in understanding its effects on wildlife. Although it is usually inappropriate to draw statistical inference from studies whose methodology is not standardized, there is still substantial information that can be gained by comparison of data in these studies. Our work highlights both the strengths and weaknesses of this approach and it also identifies a number of species that may prove to be of particular concern to managers because of the frequency with which they are found dead at renewable energy facilities within the DRECP area.

## Supporting information

S1 Table Fatality data obtained from monitoring reports and SPUT datasets from solar energy facilities within the desert renewable energy plan area. (XLSX)

S2 Table Fatality data obtained from monitoring reports and SPUT datasets from wind energy facilities within the desert renewable energy plan area. (XLSX)

S1 Text All reports included in analyses for fatality surveys and pre-construction use surveys. (PDF)
