# Supplementary material for: Numbers of wildlife fatalities at renewable energy facilities in a targeted development region
Source: PLoS One. 2023 Dec 15;18(12):e0295552. doi: 10.1371/journal.pone.0295552 (PMC10723682; doi:10.1371/journal.pone.0295552)
Supplement: S1 Text — (PDF) [file pone.0295552.s003.pdf]

PLOS ONE

Numbers of wildlife fatalities at renewable energy facilities in a targeted development region

Authors: T.J. Conkling, A.L. Fesnock, and T.E. Katzner

Correspondence Author e-mail and affiliation: tconkling@usgs.gov,

U.S. Geological Survey, Forest and Rangeland Ecosystem Science Center

S1 Text. All reports included in analyses for fatality surveys

Abengoa Solar. 2013. Mojave Solar Project monthly compliance report November 2013 reporting period. Prepared for Mojave Solar LLC. December 2013.

Abengoa Solar. 2014. Mojave Solar Project monthly compliance report November 2014 reporting period. Prepared for Mojave Solar LLC. December 2014.

Abengoa Solar. 2014. Mojave Solar Project monthly compliance report October 2014 reporting period. Prepared for Mojave Solar LLC. November 2014.

Abengoa Solar. 2014. Mojave Solar Project monthly compliance report August 2014 reporting period. Prepared for Mojave Solar LLC. September 2014.

Abengoa Solar. 2014. Mojave Solar Project Monthly Compliance Report July 2014 Reporting Period. Prepared for Mojave Solar LLC. August 2014.

Abengoa Solar. 2014. Mojave Solar Project monthly compliance report March 2014 reporting period. Prepared for Mojave Solar LLC. April 2014.

Abengoa Solar. 2014. Mojave Solar Project monthly compliance report October 2013 reporting period. Prepared for Mojave Solar LLC. November 2013.

Abengoa Solar. 2015. Mojave Solar Project monthly compliance report September 2015 reporting period. Prepared for Mojave Solar LLC. October 2015.

Abengoa Solar. 2015. Mojave Solar Project monthly compliance report August 2015 reporting period. Prepared for Mojave Solar LLC. September 2015.

Abengoa Solar. 2015. Mojave Solar Project monthly compliance report June 2015 reporting period. Prepared for Mojave Solar LLC. July 2015.

Abengoa Solar. 2015. Mojave Solar Project monthly compliance report April 2015 reporting period. Prepared for Mojave Solar LLC. May 2015.

Abengoa Solar. 2015. Mojave Solar Project monthly compliance report March 2015 reporting period. Prepared for Mojave Solar LLC. November 2015.

Abengoa Solar. 2015. Mojave Solar Project monthly compliance report February 2015 reporting period. Prepared for Mojave Solar LLC. May 2015.

Abengoa Solar. 2015. Mojave Solar Project monthly compliance report January 2015 reporting period. Prepared for Mojave Solar LLC. February 2015.

Abengoa Solar. 2015. Mojave Solar Project monthly compliance report December 2014 reporting period. Prepared for Mojave Solar LLC. January 2015.

Abengoa Solar. 2016. Mojave Solar Project monthly compliance report March 2016 reporting period. Prepared for Mojave Solar LLC. April 2016.

Abengoa Solar. 2016. Mojave Solar Project monthly compliance report October 2015 reporting period. Prepared for Mojave Solar LLC. November 2015.

BioResource Consultants. 2013. Spring 2013 Mortality Monitoring Report Pine Tree Wind Farm Kern County, California February - June 2013. September 2013.

Bloom PH. 2005. Fall-Spring raptor migration and winter raptor survey of the proposed PDv Wind Energy Project Kern County, California 2004-2005. Prepared for Sapphos Environmental, Inc., Pasadena, CA. October 15, 2005.

Bloom PH. 2006. Fall 2005 raptor migration study of the proposed PdV Wind Energy Project Kern County, California. Prepared for Sapphos Environmental, Inc., Pasadena, CA.

Curry & Kerlinger, L.L.C. 2007. Final report of post-construction carcass searches Kumeyaay Wind Project, Campo Indian Reservation San Diego County, California 26 January 2006–29 January 2007. Prepared for Babcock & Brown. October 2007.

EDAW AECOM, Bloom Biological I. 2009. Blythe Solar Power Project avian point count technical report Riverside County, California. Prepared for Solar Millennium, LLC and Chevron Energy Solutions. August 2009.

H. T. Harvey & Associates. 2015. Ivanpah Solar Electric Generating System avian & bat monitoring Plan 2013–2014 annual report (revised) (29 October 2013–20 October 2014. Prepared for Solar Partners I, II, and VIII. April 2015.

HELIX Environmental Planning, Inc. 2010. Ocotillo Wind Energy Project Raptor Migration Report. Prepared for Aspen Environmental Group. September 13, 2010.

Heritage Environmental Consultants, LLC. 2013. Campo Verde Solar Project bird and bat conservation strategy. Prepared for Bureau of Land Management. February 2013.

Heritage Environmental Consultants, LLC. 2016. Post-construction avian mortality monitoring report Campo Verde Solar Project Year 3 annual report (October 2015 – September 2016). December 2016.

Heritage Environmental Consultants, LLC. 2017. Post-construction avian mortality monitoring report Calipatria Solar Project Year 1 Semi-annual report (June 2016 – October 2016). May 2017.

Heritage Environmental Consultants L. 2015. Post-construction avian mortality monitoring report Campo Verde Solar Project Year 2 annual report (October 2014 – September 2015). December 2015.

Ironwood Consulting Inc. 2012. 2011 annual report for Biological Resources monitoring First Solar's Desert Sunlight Solar Farm, Riverside County. Prepared for Bureau of Land Management, Palm Springs, California. January 2012.

Ironwood Consulting Inc. 2015. 2014 Fourth quarter and final report for biological resources monitoring First Solar Desert Sunlight Solar Project, Riverside County 1 January – 31 December 2014. Prepared for Bureau of Land Management.

Lovich JE. 2015. Golden Eagle mortality at a wind-energy facility near Palm Springs, California. Western Birds **46**:76–80.

Morrison ML. 2004. Proposed Pine Tree Wind development project: A review of potential avian wildlife-wind development impacts. Prepared for EDAW, Inc., June 22, 2004.

Ocotillo Express LLC. 2012. Avian and bat protection for the Ocotillo Wind Energy Facility. Prepared for Ocotillo Express LLC. February 2012.

State Energy Resources Conservation and Development Commission, Western EcoSystems Technology, Inc. 2004. Avian monitoring and risk assessment at the Tehachapi Pass Wind Resource Area period of performance: October 2, 1996 – May 27, 1998. Prepared for National Renewable Energy Laboratory. September 2004.

Tetra Tech. 2014. Bird and bat conservation strategy. Modified Blythe Solar Power Project Eastern Riverside County, California. Prepared for NextEra Blythe Solar Energy Center, LLC. May 2014.

Tetra Tech. 2014. Fall 2013 and Winter 2013/2014 avian survey report. Modified Blythe Solar Power Project Riverside County, CA. Prepared for NextEra Blythe Solar Energy Center, LLC. May 2014.

Tetra Tech. 2014. Genesis Solar Revised.3.17.2014 bird and bat conservation strategy (Avian Plan) Genesis Solar Energy Project Eastern Riverside County, California. Prepared for Genesis Solar, LLC. March 2014.

Tetra Tech, Karl AE. 2010. 2009 Winter avian Point Count and Burrowing Owl Survey Report Genesis Solar Energy Project, Riverside County, CA. Prepared for Genesis Solar, LLC. April 2010.

Weller TJ, Domschke C. 2015. Post-construction fatality monitoring at the Manzana Wind Plant Kern County, California Two Year Report. Prepared for Iberdrola Renewables, Portland, Oregon. August 2015.

Western EcoSystems Technology, Inc. 2009. Avian and bat fatality study Dillon Wind-Energy Facility Riverside County, California Final report March 26, 2008 – March 26, 2009. Prepared for Iberdrola Renewables. June 3, 2009.

Western EcoSystems Technology, Inc. 2010. Avian and bat fatality study at the Alite Wind-Energy Facility Kern County, California Final report June 15, 2009 – June 15, 2010. Prepared for CH2M HILL. August 24, 2010.

Western EcoSystems Technology, Inc. 2011. Avian baseline studies at the Alta East Wind Resource Area Kern County, California Final report June 10, 2010–June 1, 2011. Prepared for CH2M Hill. July 13, 2011.

Western EcoSystems Technology, Inc. 2011. Avian baseline studies for the North Sky River Wind Energy Project Kern County, California Final report May 18, 2010–May 26, 2011. Prepared for CH2M HILL. July 7, 2011.

Western EcoSystems Technology, Inc. 2012. Avian and bat mortality monitoring at the Alta-Oak Creek Mojave Project Kern County, California Final report for the first year of operation March 22, 2011–June 15, 2012. Prepared for Alta Windpower Development, LLC. September 12, 2012.

Western EcoSystems Technology, Inc. 2014. Post-construction studies for the Mustang Hills and Alta VIII wind energy facilities Kern County, California Final report for the first year of Operation July 2012– October 2013. Prepared for EverPower Wind Holdings, Inc. and Brookfield Renewable Energy Group. February 28, 2014.

Western EcoSystems Technology, Inc. 2014. Post-construction studies for the North Sky River Wind Energy Facility Final report for the First Year of Operation January 2013–January 2014. Prepared for North Sky River Energy LLC. March 31, 2014.

Western EcoSystems Technology, Inc. 2015. Post-construction avian and bat mortality monitoring Alta X, LLC Kern County, California Final report for the first year of operation March 2014–March 2015. Prepared for Alta Wind X, LLC. July 10, 2015.

Western EcoSystems Technology, Inc. 2015. Post-construction monitoring at the Genesis Solar Energy Project Riverside County, California 2015 Spring report. Prepared for Genesis Solar, LLC. December 9, 2015.

Western EcoSystems Technology, Inc. 2015. Post-construction studies for the North Sky River Wind Energy Facility Kern County, California Final Report for the Second Year of Operation January 2014 – January 2015. Prepared for North Sky River Energy LLC. April 22, 2015.

Western EcoSystems Technology, Inc. 2016. Bird and bat conservation strategy Blythe Solar Power Project Riverside County, California. Prepared for NextEra Blythe Solar Energy Center, LLC. May 2, 2016.

Western EcoSystems Technology, Inc. 2016. Ivanpah Solar Electric Generating System avian & bat monitoring Plan 2014-2015 annual report and 2 year comparison 21 October 2014 – 20 October 2015. Prepared for Solar Partners I, II, and VIII. June 2016.

Western EcoSystems Technology, Inc. 2016. Post-construction monitoring at Genesis Solar Energy Project, Riverside County, California 2015 Summer Quarterly Interim report. Prepared for Genesis Solar LLC. February 2, 2016.

Western EcoSystems Technology, Inc. 2016. Post-construction monitoring at Genesis Solar Energy Project, Riverside County, California 2015 Fall Quarterly Interim report. Prepared for Genesis Solar, LLC. May 6, 2016.

Western EcoSystems Technology, Inc. 2016. Post-construction monitoring at the Blythe Solar Power Project Riverside county, California 2016 Spring Quarterly Interim report. Prepared for NextEra Blythe Solar Energy Center, LLC. July 18, 2016.

Western EcoSystems Technology, Inc. 2016. Post-construction studies for the Mustang Hills and Alta VIII wind energy facilities Kern County, California Draft report for the third year of operation September 2014 – September 2015. Prepared for EverPower Wind Holdings, Inc. and Brookfield Renewable Energy Group. February 22, 2016.

Western EcoSystems Technology, Inc. 2016. Post-construction studies for the North Sky River Wind Energy Facility Kern County, California Final Report for the Third Year of Operation January 2015 – January 2016. Prepared for North Sky River Energy LLC. June 17, 2016.

Western EcoSystems Technology, Inc. 2017. Post-construction studies for the North Sky River Wind Energy Facility Kern County, California Final report for the Fourth Year of Operation January 2016 - January 2017. Prepared for North Sky River Wind Energy, LLC. May 16, 2017.

Western EcoSystems Technology, Inc., California Energy Commission, Pacific Southwest Research Station. 2002. Avian monitoring and risk assessment at Tehachapi Pass and San Gorgonio Pass Wind Resource Areas, California: Phase 1 preliminary results. Proceedings of National Avian-Wind Power Planning Meeting III, San Diego, California, May 1998. Prepared for the Avian Subcommittee of the National Wind Coordinating Committee by LGL Ltd., King City, Ontario.

Western EcoSystems Technology, Inc., State Energy Resources Conservation and Development Commission. 2005. Avian monitoring and risk assessment at the San Gorgonio Wind Resource Area Phase I Field Work: March 3, 1997–May 29, 1998, Phase II Field Work: August 18, 1999–August 11, 2000. Prepared for National Renewable Energy Laboratory. August 2005.

Western EcoSystems Technology I. 2016. Post-Construction Avian and Bat Mortality Monitoring at the Alta X Wind Energy Project, Kern County, California Final Report for the Second Year of Operation April 2015 – April 2016. Prepared by Western Ecosystems Technology, Inc., 22 July 2016.

Western EcoSystems Technology I. 2017. Post-Construction Avian and Bat Mortality Monitoring at the Alta X Wind Energy Project, Kern County, California Report for the

Third Year of Operation, April 2016 - April 2017. Prepared by Western Ecosystems Technology, Inc., 14 July 2017.

Western EcoSystems Technology I. 2018. Eagle Fatality Compliance Monitoring for the Alta X Wind Energy Project Kern County, California Report for the First Year of Monitoring Under the Alta Wind X, LLC Incidental Take Permit for Golden Eagles May 2017 -May 2018.

Western EcoSystems Technology I. 2019. Eagle Fatality Compliance Monitoring for the Alta X Wind Energy Project Kern County, California Report for the Second Year of Monitoring Under the Alta Wind X, LLC Incidental Take Permit for Golden Eagles May 2018–May 2019. 10 July 2019.

Western EcoSystems Technology I. 2019. Third Year of Post-Construction Wildlife Studies at the Ocotillo Express Wind Energy Facility Imperial County, California March 21, 2018 - March 16, 2019 Confidential-Draft Report.
